# Supplementary material for: Work–Family Interaction, Self-Perceived Mental Health and Burnout in Specialized Physicians of Huelva (Spain): A Study Conducted during the SARS-CoV-2 Pandemic
Source: Int J Environ Res Public Health. 2022 Mar 21;19(6):3717. doi: 10.3390/ijerph19063717 (PMC8955258; doi:10.3390/ijerph19063717)
Supplement: Supplementary file 1 [file ijerph-19-03717-s001.zip › ijerph-1607518-supplementary/ijerph-1607518-supplementary Table S1.pdf]

# EVALUATION INSTRUMENTS

| Sociodemographic information |
|---|

- Sex _______________
- Age _______________
- Marital status _______________
- Parenthood ____________________
- Service time_________________
- Workplace_____________________________
- Have you had contact with any COVID-19 situation in your work environment? _________

| Maslach Burnout Inventory (MBI) |
|---|

You must respond to each of the sentences by expressing the frequency with which you have that feeling, meaning: 0="Never" and 6="Every day"

| **Questions** | **Never** | **Few times a year** | **Once a month or less** | **A few times a month or less** | **Once a week** | **Few times a week** | **Every day** |
|---|---|---|---|---|---|---|---|
| **Item No.** | **0** | **1** | **2** | **3** | **4** | **5** | **6** |
| **1.- I feel emotionally drained at my job** | | | | | | | |
| **2.- I feel tired at the end of the working day** | | | | | | | |
| **3.- I feel fatigued when I get up in the morning and have to go to work** | | | | | | | |
| **4.- I easily understand how patients feel** | | | | | | | |
| **5.- I treat some patients as if they were impersonal objects** | | | | | | | |
| **6.- Working all day with many people is an effort** | | | | | | | |

| | | | | | | | |
|---|---|---|---|---|---|---|---|
| **7.- I deal very effectively with patient problems** | | | | | | | |
| **8.- I feel "burned out" by my work** | | | | | | | |
| **9.- I believe that I positively influence people's lives with my work** | | | | | | | |
| **10.- I've become more insensitive to people since I've been in this profession** | | | | | | | |
| **11.- I am concerned that this job will harden me emotionally** | | | | | | | |
| **12.- I feel very active** | | | | | | | |
| **13.- There are times when I feel frustrated in my work** | | | | | | | |
| **14.- I think I'm working too hard** | | | | | | | |
| **15.- I don't really care what happens to my patients** | | | | | | | |
| **16.- Working directly with people causes me stress** | | | | | | | |
| **17.- I can easily create a relaxed atmosphere with my patients** | | | | | | | |
| **18.- I feel stimulated after working with my patients** | | | | | | | |

| | | | | | | | |
|---|---|---|---|---|---|---|---|
| **19.- I have achieved many useful things in my profession** | | | | | | | |
| **20.- Lately I feel finished** | | | | | | | |
| **21.- In my work I deal with emotional problems very calmly** | | | | | | | |
| **22.- I feel that patients blame me for some of their problems** | | | | | | | |

**Survey Work-Home Interaction - Nijmegen (SWING)**

Below you will find a series of situations about how the work and personal spheres are related. Please indicate how often you have experienced each of the following situations during the last six months. Mark with a cross the box that best describes your opinion according to the following response scale: 0="Never", 1="Sometimes", 2="Often" and 3="Always".

| **Questions** | **Never** | **Sometimes** | **Often** | **Always** |
|---|---|---|---|---|
| **Item No** | **0** | **1** | **2** | **3** |
| **1.- I feel irritable at home because my work is very tiring** | | | | |
| **2.- It is difficult for me to attend to my domestic obligations because I am constantly thinking about my work** | | | | |
| **3.- I have to cancel plans with my partner/family/friends due to work commitments** | | | | |
| **4.- My work schedule makes it difficult to attend to my domestic obligations** | | | | |
| **5.- I do not have enough energy to do leisure activities with my partner/family/friends due to my work** | | | | |

| | | | | |
|---|---|---|---|---|
| **6.- I have to work so much that I don't have time for my hobbies** | | | | |
| **7.- My work obligations make it difficult for me to relax at home** | | | | |
| **8.- My job takes away time that I would have liked to spend with my partner/family/friends** | | | | |
| **9.- The situation at home makes me so irritable that I take my frustration out on my co-workers** | | | | |
| **10.- I find it difficult to concentrate on my work because I am preoccupied with domestic matters** | | | | |
| **11.- Problems with my partner/family/friends affect my work performance** | | | | |
| **12.- The problems I have with my partner/family/friends make me not want to work** | | | | |
| **13.- After a pleasant day or week of work, I feel in a better mood to do activities with my partner/family/friends** | | | | |
| **14.- I perform my domestic duties better thanks to skills I have learned on the job** | | | | |
| **15.- I duly fulfill my responsibilities at home because in my work I have acquired the ability to commit to things** | | | | |
| **16.- Having to organize my time at work has made me learn to organize my time at home** | | | | |
| **17.- I am able to interact better with my partner/family/friends thanks to the skills I have learned at work** | | | | |
| **18.- After spending a fun weekend with my partner/family/friends, I find my job more enjoyable** | | | | |
| **19.- I take my work responsibilities very seriously because at home I have to do the same** | | | | |
| **20.- I duly fulfill my work responsibilities because at home I have acquired the ability to commit to things** | | | | |

| | | | | |
|---|---|---|---|---|
| **21.- Having to organize my time at home has made me learn to better organize my time at work** | | | | |
| **22.- I have more self-confidence at work because my life at home is well organized** | | | | |

**GHQ-12**

Next, you are asked a series of questions about how your health has been in the PAST few weeks. Answer by placing a cross to the left of the answer that best fits your experience.

1. Have you been able to concentrate well on what you are doing?
___ 0. Better than usual.
___ 1. Same as usual.
___ 2. Less than usual.
___ 3. Much less than usual.

2. Have your worries made you lose a lot of sleep?
___ 0. Not at all.
___ 1. Same as usual.
___ 2. More than usual.
___ 3. Much more than usual.

3. Have you felt that you are playing a useful role in life?
___ 0. More than usual.
___ 1. Same as usual.
___ 2. Less than usual.
___ 3. Much less than usual.

4. Have you felt capable of making decisions?
___ 0. More capable than usual.
___ 1. Same as usual.
___ 2. Less capable than usual.
___ 3. Much less capable than usual.

5. Have you constantly felt overwhelmed and stressed?
___ 0. Not at all.
___ 1. Same as usual.
___ 2. More than usual.
___ 3. Much more than usual.

6. Have you felt that you cannot overcome your difficulties?
___ 0. Not at all.
___ 1. Same as usual.
___ 2. More than usual.
___ 3. Much more than usual.

7. Have you been able to enjoy your normal daily activities?
___ 0. More than usual.
___ 1. Same as usual.
___ 2. Less than usual.
___ 3. Much less than usual.

8. Have you been able to deal adequately with your problems?
___ 0. More capable than usual.
___ 1. Same as usual.
___ 2. Less capable than usual.
___ 3. Much less capable than usual.

9. Have you felt unhappy or depressed?
___ 0. Not at all.
___ 1. No more than usual.
___ 2. More than usual.
___ 3. Much more than usual.

10. Have you lost confidence in yourself?
___ 0. Not at all.
___ 1. No more than usual.
___ 2. More than usual.
___ 3. Much more than usual.

11. Have you thought that you are a worthless person?
___ 0. Not at all.
___ 1. No more than usual.
___ 2. More than usual.
___ 3. Much more than usual.

12. ¿Se siente razonablemente feliz considerando todas las circunstancias?
___ 0. Más feliz que lo habitual.
___ 1. Igual que lo habitual.
___ 2. Menos feliz que lo habitual.
___ 3. Mucho menos feliz que lo habitual.

12. Are you reasonably happy considering all the circumstances?
___ 0. Happier than usual.
___ 1. Same as usual.
___ 2. Less happy than usual.
___ 3. Much less happy than usual.

Thank you very much for your help. If you have any suggestion you can write it in this section. If you want to know the results of your questionnaire or have any questions, you can leave us your email and we will send you the answer as soon as possible. A cordial greeting.

________________________________________________________________________
________________________________________________________________________
________________________________________________________________________
________________________________________________________________________
________________________________________________________________________
______________
